# 

**Published:** 1788

**Authors:** 


### CONTENTS.

Page

I. *OF the epidemic Catarrh of the Year* 1788. *By* Samuel Foart Simmons, *M. D. F. R. S.* — — — 335

II. *Of the epidemic Catarrh of the Year* 1788. *By* George Bew, *M. D. Phyſician at Mancheſter.* — — — 354

III. *An Account of the ſucceſsful Employment of Catgut in a Caſe of Fiſtula in Perinæo. By Mr.* G. Wilkinſon, *Surgeon at Sunderland, and Member of the Royal College of Surgeons of Edinburgh.* — 378

IV. *Caſe of a Suppreſſion of Urine, which terminated fatally; with an Account of the Appearances on Diſſection. By Mr.* James Stevenſon, *Surgeon at Egham, in Surry.* 382

V. *An Account of a Caſe of Amauroſis cured by Electricity. By Mr.* Miles Partington. 389

VI. *An Account of the Preparation and Uſe of the Phoſphorated Soda; being an Abſtract of a Paper on the Subject, inſerted in the Journal de Phyſique for Auguſt,* 1788, *by* George Pearſon, *M. D. Member of the College of Phyſicians, Phyſician to St. George's Hoſpital,*

Page

*Hospital, and Lecturer on Physic and Chemistry in London, with considerable Additions; by the Author.* — — — 393

VII. *An Account of the Effects of the Astragalus Exscapus* Linn. *in the Cure of the Venereal Disease; in a Letter from* A. Crichton, *M. D. to* Chr. Girtanner, *M. D. corresponding Member of the Royal Society at Gottingen. Translated from the German.* 405

CATALOGUE *of* BOOKS, — 415

INDEX, — — — — 434

